# Design and baseline characteristics of the ParkFit study, a randomized controlled trial evaluating the effectiveness of a multifaceted behavioral program to increase physical activity in Parkinson patients

**DOI:** 10.1186/1471-2377-10-70

**Published:** 2010-08-19

**Authors:** Marlies van Nimwegen, Arlène D Speelman, Katrijn Smulders, Sebastiaan Overeem, George F Borm, Frank JG Backx, Bastiaan R Bloem, Marten Munneke

**Affiliations:** 1 Radboud University Nijmegen Medical Centre (RUNMC); Nijmegen Centre for Evidence Based Practice (NCEBP), Department of Neurology, Nijmegen, The Netherlands; 2 RUNMC; Donders Institute for Brain, Cognition and Behavior; Department of Neurology, Nijmegen, The Netherlands; 3 RUNMC; Departments of Rehabilitation and Allied Health Occupations, Nijmegen, The Netherlands; 4 HAN University of Applied Sciences; Nijmegen, The Netherlands; 5 RUNMC; Department of Epidemiology, Biostatistics and HTA, Nijmegen, The Netherlands; 6 University Medical Center Utrecht; Department of Rehabilitation, Nursing Science and Sport, Utrecht, The Netherlands; 7 RUNMC; Nijmegen Centre for Evidence Based Practice (NCEBP), Scientific Institute for Quality of Healthcare, Nijmegen, The Netherlands

## Abstract

**Background:**

Many patients with Parkinson's disease (PD) lead a sedentary lifestyle. Promotion of physical activities may beneficially affect the clinical presentation of PD, and perhaps even modify the course of PD. However, because of physical and cognitive impairments, patients with PD require specific support to increase their level of physical activity.

**Methods:**

We developed the ParkFit Program: a PD-specific and multifaceted behavioral program to promote physical activity. The emphasis is on creating a behavioral change, using a combination of accepted behavioral motivation techniques. In addition, we designed a multicentre randomized clinical trial to investigate whether this ParkFit Program increases physical activity levels over two years in sedentary PD patients. We intended to include 700 sedentary patients. Primary endpoint is the time spent on physical activities per week, which will be measured every six months using an interview-based 7-day recall.

**Results:**

In total 3453 PD patients were invited to participate. Ultimately, 586 patients - with a mean (SD) age of 64.1 (7.6) years and disease duration of 5.3 (4.5) years - entered the study. Study participants were younger, had a shorter disease duration and were less sedentary compared with eligible PD patients not willing to participate.

**Discussion:**

The ParkFit trial is expected to yield important new evidence about behavioral interventions to promote physical activity in sedentary patients with PD. The results of the trial are expected in 2012.

**Trial registration:**

http://clinicaltrials.gov (nr NCT00748488).

## Background

Parkinson's disease (PD) is a progressive neurological disorder characterized by both motor symptoms (such as bradykinesia and postural instability) and non-motor symptoms (such as depression and cognitive impairment)[1]. Both motor and non-motor symptoms can result in reduced physical activity[2,3].

Observations in non-parkinsonian populations suggest that participating in regular physical activity has preventive effects (e.g. cardiovascular events, diabetes mellitus, dementia)[4-6] and positive symptomatic effects (on depression[7], sleep disturbances[8], health-related quality of life)[9]. Studies in PD patients concluded that brief physical therapy interventions can improve flexibility, balance and muscle strength[10,11]. In addition, preclinical evidence in animals with experimental parkinsonism raised the possibility that physical activity may directly alter the neurodegenerative process[12,13].

A critical question remains how PD patients can be stimulated best to achieve an enduring increase in their physical activities in daily life, in order to prevent co-morbid complications and to improve symptoms.

Simply informing subjects about the health benefits of physical activity is not enough to attain a sustained behavioral change. The challenges to induce a lasting change in exercise behavior are particularly great for neurological patients. To change lifestyle, behavioral programs should focus on appropriate supervision, social support from spouses and caregivers, and the individual's preferences and needs[14-16]. Achieving an enduring behavioral change also calls for specific strategies such as goal setting, problem-solving techniques and motivational interviewing[14,16,17]. Physical activity promoting programs including such elements were effective in sedentary people[15], patients with chronic heart failure[16], and patients with COPD[18].

Stimulated by these observations, we developed the ParkFit program: a multifaceted intervention to promote physical activity in sedentary patients with PD. In addition, we developed the ParkFit trial to investigate whether this program affords increased physical activity levels that persist for two years. The trial will also search for possible health benefits and risks of increased physical activity. Here, we describe the study design and baseline characteristics of this ParkFit trial.

## Methods

### Study Design

**Figure 1:**
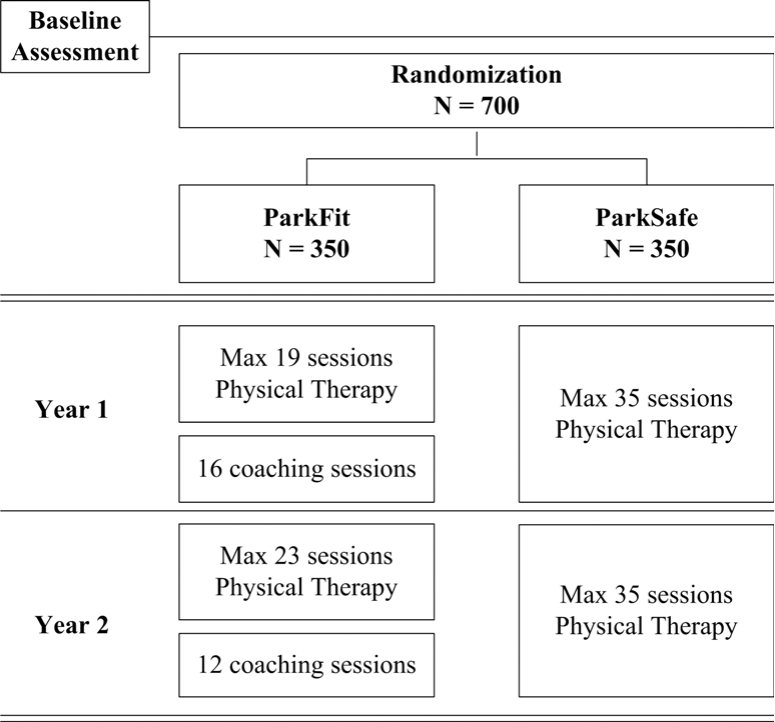
**Design of the ParkFit study**.

The ParkFit trial is a multicentre, randomized controlled trial comparing two arms: physical therapy with specific emphasis on promoting a physically active lifestyle (ParkFit Program); and matched physical therapy with specific emphasis on safety and quality of performing daily activities (ParkSafe Program) (Figure 1). Trial duration is two years. Full ethical approval has been granted for the study (CMO Regio Arnhem-Nijmegen). The study is registered at clinicaltrials.gov (nr NCT00748488).

### Patients

We started with all patients who visited their neurologist in 2007, 2008 or 2009 in 32 participating community hospitals. Eligibility criteria were: (a) PD, according to the UK Brain Bank Criteria[19]; (b) age between 40 and 75 years; (c) sedentary lifestyle defined as: < 3 times a week vigorous-intensity physical activity for < 60 minutes; or < 3 times a week moderate-intensity physical activity for < 150 minutes);[20] (d) Hoehn and Yahr ≤ 3. Exclusion criteria were: (a) unclear diagnosis (no gratifying and sustained response to dopaminergic therapy); (b) MMSE < 24); (c) unable to complete Dutch questionnaires; (d) severe co-morbidity interfering with daily functioning; (e) daily institutionalized care; and (f) deep brain surgery. Informed consent was obtained before the first assessment.

### The Intervention

After baseline assessment, patients were randomly assigned to the ParkFit or ParkSafe Program. In both groups, patients receive high quality physical therapy: both interventions are delivered exclusively by experienced therapists who participate in the Dutch ParkinsonNet[21,22]. Patients in both treatment arms are offered an equal maximum number of treatment sessions (i.e. 35 sessions of 30 minutes a year; Table 1). Therapists contact patients at least every six months to investigate if there are new aims.

**Table 1:** The ParkFit and the ParkSafe program

	ParkFit	ParkSafe
**Intensity** **Year 1**	Maximum of 19 physical therapy sessions based on problems and disabilities as perceived by each individual patient; the systematic way of tailoring goals is described in the evidence-based guideline for physical therapy in PD	Maximum of 35 physical therapy sessions based on problems and disabilities as perceived by each individual patient; the systematic way of tailoring goals is described in the evidence-based guideline for physical therapy in PD
	16 coaching sessions to identify and focus on individual beliefs and aims to promote a physically active lifestyle	
**Intensity** **Year 2**	Maximum of 23 physical therapy sessions based on problems and disabilities as perceived by each individual patient	Maximum of 35 physical therapy sessions based on problems and disabilities as perceived by each individual patient
	12 coaching sessions to identify and focus on individual beliefs and aims to promote a physically active lifestyle	
**Specific Elements**	ParkFit Brochure: • *Education about benefits of physical therapy* • *Identifying aims of physical therapy* • *Education about the benefits of physical activity* • *Identifying barriers to engage physical activity* • *Setting goals* • *Recruiting social support* • *Sign a health contract to support patients in initiating and maintaining* *physical activities* • *A logbook to describe and monitor the specific goals*	ParkSafe Brochure: • *Education about benefits of physical therapy* • *Identifying aims of physical therapy* • *Education about the importance of safety when performing* *daily activities*
	Physical therapist: who treat the patient in order to obtain the aims of the individual projected treatment plan Personal Activity Coach: who guide patients towards a more active lifestyle	Physical therapist: who treat the patient in order to obtain the aims of the individual projected treatment plan
	Goal setting: creating goals to increase the level of physical activity in order to obtain the half- year-goals as formulated in the health contract; goals will be evaluated as well as experienced barriers and possible solving techniques	
	Ambulatory Activity Monitor: gives visual feedback about the level of physical activity during the day	
	Bi-annual newsletter: specific information about physical activity, general information about Parkinson's disease, and general entertainment in order to facilitate compliance	Bi-annual newsletter: specific information about physical therapy, general information about Parkinson's disease, and general entertainment in order to facilitate compliance

#### ParkFit Program

Widely used behavioral change techniques, with demonstrated effectiveness[16,18] and based on models of behavioral change[14,17], are combined in the ParkFit Program to stimulate patients to increase their physical activity levels.

##### 1) Brochure ParkFit

Patients receive a brochure covering specific strategies to promote a behavioral change. These strategies include: education about the benefits of physical activity, advice about suitable activities, identifying and overcoming any perceived barriers to engage in physical activity, setting goals, and recruiting social support[14,23,24]. Part of the educational workbook is a health contract: a written agreement signed by the patient and physiotherapist to support them in initiating and maintaining physical activities[25]. A logbook monitors the specific goals.

##### 2) Personal Activity Coach

Physical therapists serve as personal activity coaches who guide patients towards a more active lifestyle, during specific coaching sessions. Their task is to educate patients about the beneficial effects of physical activity. Patients are additionally stimulated to participate in group exercise to experience the beneficial effects of physical activity and to receive social support from fellow patients[26]. For safety reasons, all patients are encouraged to receive a preventive sports medical screening.

##### 3) Goal setting

Patient and coach create activity goals in order to obtain the 6-month-goals (as formulated in the health contract). Goals have to be realistic, concrete and individualized and have to be formulated in a systematic way, based on behavioral change theories[25].

##### 4) Ambulatory Activity Monitor with visual feedback

Patients receive a personal ambulatory monitor with automated visual feedback showing the amount of actually delivered daily physical activity, recorded by a triaxial accelerometer[27,28]. Additionally, a personalized website shows the activity history[27]. Previous work showed that feedback from pedometers increases physical activity levels in COPD patients[29], sedentary workers[30] and patients with diabetes mellitus[31].

##### 5) Physical therapy

The ParkFit Program also includes a maximum of 19 physical therapy sessions in year 1 and 23 in year 2. Based on individual disabilities, therapist and patient jointly formulate treatment aims based on the evidence-based guideline of physical therapy for PD[32].

#### ParkSafe Program

The ParkSafe Program includes physical therapy interventions from the physical therapy guideline for PD[32] to stimulate patients to move more safely, e.g. by improving the quality of transfers, but without explicit emphasis on reaching a physically active lifestyle.

##### 1) Brochure ParkSafe

Patients receive a brochure with information about the benefits of physical therapy[32]. Specific emphasis is given to the importance of safety when performing daily activities.

##### 2) *Physical therapy*

Patients receive an individualized physical therapy program. We maximized the total number of sessions at 35/year, to avoid large differences in number of treatment sessions between the two arms (patients in the ParkFit arm also receive 35 annual sessions: 19 physiotherapy plus 16 coach sessions). 35 sessions is considered sufficient for patients in Hoehn and Yahr stage ≤ 3. Physical therapist and patient jointly formulate the aims of the projected treatment plan, based on individual problems and disabilities. The aims of the physical therapy sessions in both treatment arms are derived from the guideline for physical therapy in PD.

### Implementation

#### Training for physical therapists

All participating physical therapists were specifically trained to treat patients in both treatment arms and informed about the aim of the study. Special attention was given to models of behavioral change,[14,17] to specific strategies of coaching sedentary patients,[15,33] and to the technique of setting realistic, concrete and individualized goals[25]. Throughout the trial, therapists continuously register the individual treatment sessions.

### Outcome measures

#### Baseline characteristics

Blood pressure, height, body weight, education and employment are assessed at baseline as well as alcohol use, smoking history and lifetime physical activity[34]. Participants in the ParkFit Program also completed a questionnaire about attitude, social support and self-efficacy towards physical activity.

#### Primary endpoint: level of physical activity

Primary endpoint is the level of physical activity, as measured with a 7-day recall, based on an interview-based physical activity questionnaire, the LAPAQ[35]. Patients are asked to list their daily amount of activity (frequency and duration), so total time spent on physical activity (in hours per day) will be calculated. A MET-value will be used to calculate the number of kilocalories spent per day per kilogram of body weight[36]. The LAPAQ is completed during a face-to-face interview (at baseline, 12 and 24 months) and at additional time points by telephone (6 and 18 months). We assume that patients will increase their level of physical activity during the first months of intervention and then maintain this level. Therefore, main endpoint is the level of physical activity during the entire follow-up period (i.e. the mean of 6, 12, 18 and 24 months).

#### Secondary endpoints (Table 2)

**Table 2:** An overview of patient assessments

	Baseline	6 months	12 months	18 months	24 months
	*Visit &* *Questionnaires*	*Questionnaires*	*Visit &* *Questionnaires*	*Questionnaires*	*Visit &* *Questionnaires*
**Physical Activity**					
*LAPAQ*	x	x	x	x	x
*Activity Monitor*	x	x	x	x	x
*Activity Diary*	x	x	x	x	x
**Physical Fitness**					
*6 MWT*	x		x		x
*Åstrand-Ryhming test*	x		x		x
**Quality of Life**					
*PDQ-39*	x	x	x	x	x
**Health Effects**					
*UPDRS III, motor function*	x		x		x
*Nine hole peg board test*	x		x		x
*Timed up and go test*	x		x		x
*DXA*	x				
*SCOPA-sleep*	x	x	x	x	x
*HADS*	x	x	x	x	x
*FSS*	x	x	x	x	x
Cognitive testing battery*	x		x		x
*PD medication*	x	x	x	x	x
*Medical costs & EQ-5D*	x	x	x	x	x
*Number of falls (monthly)*	x	x	x	x	x
**Determinants**					
*Blood pressure*	x		x		x
*Height*	x		x		x
*Body weight*	x		x		x
*Education*	x				
*Employment*	x				
*Alcohol use*	x		x		x
*Smoking*	x		x		x
*Lifetime physical activity*	x				
Attitude, SS & SE**	x				

* Including tests for spatial working memory[49], *intra- and extra-dimensional shift performance*[50,51], *paired associate learning performance*[50], *phonemic and semantic word fluency*[52], *and complex figure drawing*[53]

** Only patients in the ParkFit Program

Secondary measures include: (a) physical fitness, measured with the six minute walk test (6MWT)[37]; (b) quality of life, measured with the PDQ-39[38]; and (c) level of physical activity in time and kilocalories per week, measured with the same tri-axial accelerometer that is used as feedback-tool in the ParkFit Program[28]. The level of physical activity is additionally measured with a physical activity diary.

#### Additional measures

Patients who increased their amount of physical activity will be compared with patients unable to achieve this, to assess specific health consequences. Disease progression (UPDRS motor section [39]; 9-hole peg board test[40]), mobility (Timed Up and Go test[41]), quality of sleep (SCOPA-sleep[42]), anxiety and depression (HADS[43]), fatigue (Fatigue Severity Scale[44]), and cognitive functioning (Table 2 for test battery) are assessed. Additionally, physical fitness is measured with the Åstrand-Ryhming test[45]. Bone mineral density (dual energy X-ray absorptiometry, DXA) is determined in a subgroup of 300 patients. PD medication and medical costs (combined with the EQ-5D[46]) are assessed, as well as the number of falls (as an index of safety). Patients are asked whether their falls occurred during exercise and about the consequences of falls (e.g. injuries). Information about other adverse events is collected systematically at each physical assessment.

#### Blinding

To avoid bias due to more positive expectations of patients towards the outcomes of the ParkFit Program, patients were initially informed about the fact that there are two intervention groups, each with a beneficial intervention. To ensure blinding during assessments, patients are assessed by trained assessors who are unaware of group allocation. Patients are explicitly asked to not share their experiences with the program during the assessments.

#### Sample size calculation

Based on the following power considerations, we aimed to include a total of 700 patients. In a small observational study on physical activity in PD, patients scored 45% less on the LAPAQ compared to controls (unpublished data). The coefficient of variation was 110%. Based on a difference of 15% (with coefficient of variation of 110%) between both treatment arms, the study will have at least 80% power (when the correlation between baseline and follow-up measurements is at least 0.50 and when the correlation between the various follow-up measurements is at most 0.85). This is also the power when the correlations are at least 0.60 and at most 0.95, respectively. The power is based on two-sided 95% confidence intervals. We assumed that patients would take part in exercise groups with on average eight participants and that the corresponding ICC would be 0.1. Based on a previous trial of physical therapy for PD involving the national ParkinsonNet networks[21], we expect a drop-out rate of 10%.

#### Randomization

A minimization algorithm is used to randomize patients, with the factors region, Hoehn & Yahr stage, age, gender and current level of physical activity.

#### Statistical analyses

All participants who really started with their program, will be included in the primary analysis. The results after 6, 12, 18 and 24 months will be evaluated using a linear mixed model with random nested factors 'patient' and 'exercise group'. Fixed factors will be treatment arm, LAPAQ score at baseline, month, month*treatment (interaction), and the factors region, H&Y stage, age, gender and bone density assessment. In an additional analysis, the influence of H&Y stage, age, gender and level of previous sports activities on the success of the treatment will be evaluated by including the interaction terms between treatment and each of these variables in the model. Multiple imputation analyses will be used to evaluate the impact of missing values on the outcome. Throughout, 95% confidence intervals will be calculated.

## Results

### Inclusion procedure

**Figure 2:**
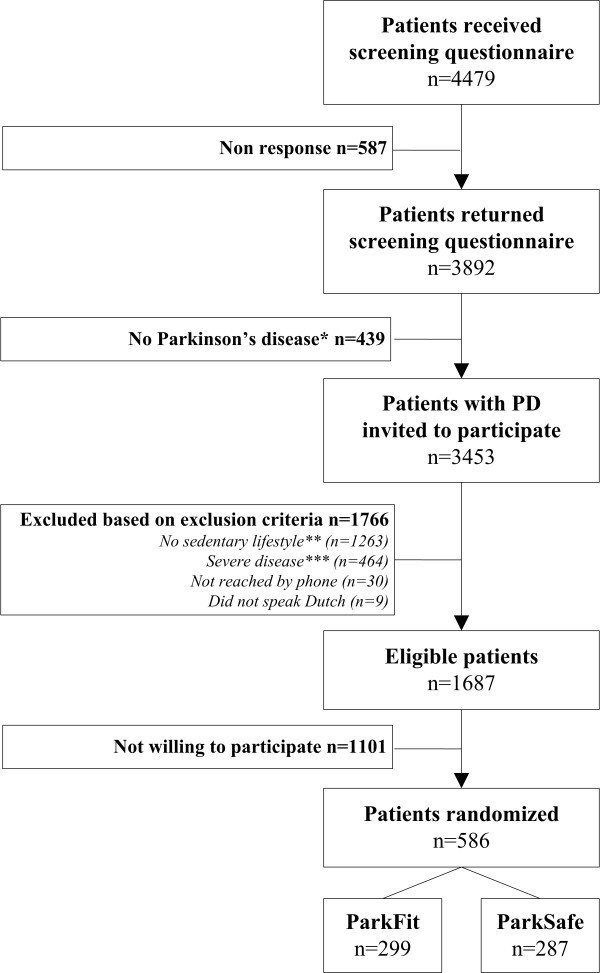
**Flow chart of the inclusion procedure**. * No Parkinson's disease = patient is diagnosed with parkinsonism or patient declared to have no gratifying and sustained response to dopaminergic therapy; ** No sedentary lifestyle = > 3 times a week vigorous-intensity physical activity > 60 minutes; or > 3 times a week moderate-intensity physical activity > 150 minutes; *** Severe disease = H&Y > III; MMSE < 24; severe co-morbidity interfering with daily functioning; use of daily care in an institution; or deep brain stimulation.

Selection of patients ran from September 2008 to January 2010. A total number of 4479 patients received a screening questionnaire; 587 (13.1%) did not respond, 439 (9.8%) were excluded because there was doubt about the diagnosis (Figure 2). After invitation for participation, 1766 patients were excluded based on our exclusion criteria, and 1101 eligible patients were excluded because they were not willing to participate. Finally, 586 patients signed informed consent. The number of enrolled patients is less than the power calculation required. However, the power remains over 80% because only 60% of patients participates in exercise groups with an average group size of only three, whereas our power calculation assumed that all patients would participate in exercise in groups of eight patients.

### Baseline characteristics

The most relevant baseline characteristics of included patients are presented in Table 3 and compared with the characteristics of the complete cohort of PD patients and the cohort of patients who were eligible but not willing to participate. Study participants were younger, had a shorter disease duration and were less sedentary compared with eligible patients not willing to participate.

**Table 3:** Characteristics of the invited and included patients

	Complete cohort of PD patients	Eligible patients	
		*Not willing to participate*	*Willing to participate*
**N**	3453	1101	586
**Gender **(% male)	59.0	53.4	65.4
**Age **(years)	66.1 (7.2)	67.2 (7.1)	64.1 (7.6)
**Disease duration **(years)	6.2 (5.7)	6.0 (5.6)	5.3 (4.6)
**Ability to walk **(%)			
*Normal*	814 (23.7)	232 (21.3)	157 (26.8)
*Slow but independently*	1747 (50.8)	556 (51.0)	348 (59.4)
*Independently with walking aid*	605 (17.6)	302 (27.7)	81 (13.8)
*With help of someone*	112 (3.3)		
*Wheelchair bounded*	158 (4.6)		
**Level of physical activity **(min/week)	144.8 (196.7)	40.1 (61.1)	59.2 (71.9)

## Discussion

Several lines of evidence suggest that regular participation in physical activity could be important for patients with PD[47]. The ParkFit trial was designed to evaluate a multifaceted program to achieve an enduring increase in physical activity in PD patients. The intervention is based on accepted motivational and behavioral change models[14,16,17], which will now be employed for the first time in PD.

We carefully monitored the characteristics of all invited patients as well as eligible patients who were not willing to participate. The results demonstrate that among all PD patients who were invited, 64% indeed had a sedentary lifestyle. The results further demonstrate that eligible PD patients not willing to participate were on average somewhat more sedentary in comparison with the participants of the study. Should our study shows a beneficial effect of the ParkFit behavioral change program, efforts must be made to also reach out to this subgroup of sedentary patients.

A critical issue in rehabilitation studies is the choice for an appropriate control condition, and we have selected a program that emphasized safety of movement (according to evidence-based guidelines[32]), rather than the quantity of movements. Both intervention programs are matched for intensity, and are delivered by the same therapists. We have taken several measures to avoid bias between both treatment arms, rendering both groups comparable except for the focus on physical activities. Because the same therapists participate in both programs, differences in their personalities should not differ between the two treatment arms. A possible drawback is contamination. Furthermore, personal preference for a specific program can possibly introduce variation between therapists. We strive to avoid this by: (1) specific training, informing all therapists about the aim of the study and the do's and don'ts in both treatment arms. They have signed a contract and agreed to keep both programs separate. (2) The tools used in ParkFit are not freely available. Since all patients receive their own Activity Monitor and brochure, therapists cannot give these tools to patients allocated to ParkSafe. (3) During the trial, therapists are being visited and observed during one or more sessions. A standardized checklist of prescribed interventions will be completed to investigate if contamination is at play. (4) Each therapist will be interviewed, between 3 to 6 months after start of the program. The aim is to investigate how therapists put the program into practice, and to re-emphasize the do's and don'ts of both programs. (5) About every two months, the research team contacts each therapist to ask them about their individual aims in both treatment arms. Again, it is emphasized that coaching towards a more physically active lifestyle is not allowed in ParkSafe. (6) Yearly, a 'booster' session is planned for therapists to discuss possible problems and to re-emphasize the do's and don'ts.

A strong element of the ParkFit trial is the availability of our national ParkinsonNet networks[21], which allows us to administer the interventions in both treatment arms by therapists with documented experience in treating PD patients. The ParkFit trial is one of the largest and longest lifestyle intervention trials in PD, and is the first one to focus on behavioral change as an intermediate to achieve a sustained increase in physical activity levels.

The endpoints of this trial cover several complementary domains. A prerequisite is that patients will actually increase their physical activity levels. To document this, we have selected the time spent on physical activities per week as primary endpoint. We choose the LAPAQ as primary outcome measure instead of the Activity Monitor because a questionnaire covers a wider range of activities[48].

We also want to see whether physical activity affords any symptomatic relief of PD. To this end we have included a battery of additional endpoints (including quality of life) that measure possible health benefits for patients. Safety is also an issue, because physical activity may theoretically predispose patients to falls. Therefore, this will also be documented in this study. Furthermore, costs will be recorded, although we have no specific a priori reason to expect drastic increases or reductions in costs associated with the interventions of this trial.

In conclusion, the ParkFit trial is expected to yield important new knowledge about behavioral interventions for patients with PD to change their sedentary lifestyle. If the ParkFit Program shows good treatment compliance and beneficial symptomatic effects, future trials could identify which components of our multifaceted approach are most effective. In addition, positive results may have implications for different neurological disorders where beneficial effects of physical activity may be expected. The results of the ParkFit trial are scheduled for 2012.

## Competing interests

The authors declare that they have no competing interests.

## Authors' contributions

MM, BRB and FJGB wrote the grant application and supervised all project staff. MvN, ADS, GFB, FJGB, BRB, MM, and members of the ParkFit study group contributed to the research design. MvN, ADS, KS and MM participated in organization and execution of the research project. GFB was responsible for sample size calculation and statistical analysis. MvN and SO wrote first draft of the manuscript. All authors read and approved the final manuscript.

## Pre-publication history

The pre-publication history for this paper can be accessed here:

http://www.biomedcentral.com/1471-2377/10/70/prepub
